# Disc Volume Reduction with Percutaneous Nucleoplasty in an Animal Model

**DOI:** 10.1371/journal.pone.0050211

**Published:** 2012-11-27

**Authors:** Richard Kasch, Birger Mensel, Florian Schmidt, Sebastian Ruetten, Thomas Barz, Susanne Froehlich, Rebecca Seipel, Harry R. Merk, Ralph Kayser

**Affiliations:** 1 Clinic and Outpatient Clinic for Orthopedics and Orthopedic Surgery, Greifswald University Medicine, Greifswald, Germany; 2 Department of Diagnostic Radiology and Neuroradiology, Greifswald University Medicine, Greifswald, Germany; 3 Department of Spine Surgery and Pain Therapy, Center for Orthopedics and Traumatology, St Anna-Hospital Herne, Herne, Germany; 4 Department of Orthopaedics and Trauma Surgery, Asklepios Hospital Uckermark, Schwedt, Germany; 5 Department of Orthopedic Surgery, University Medical Center of Rostock, Rostock, Germany; University of California Los Angeles, United States of America

## Abstract

**Study Design:**

We assessed volume following nucleoplasty disc decompression in lower lumbar spines from cadaveric pigs using 7.1Tesla magnetic resonance imaging (MRI).

**Purpose:**

To investigate coblation-induced volume reductions as a possible mechanism underlying nucleoplasty.

**Methods:**

We assessed volume following nucleoplastic disc decompression in pig spines using 7.1-Tesla MRI. Volumetry was performed in lumbar discs of 21 postmortem pigs. A preoperative image data set was obtained, volume was determined, and either disc decompression or placebo therapy was performed in a randomized manner. Group 1 (nucleoplasty group) was treated according to the usual nucleoplasty protocol with coblation current applied to 6 channels for 10 seconds each in an application field of 360°; in group 2 (placebo group) the same procedure was performed but without coblation current. After the procedure, a second data set was generated and volumes calculated and matched with the preoperative measurements in a blinded manner. To analyze the effectiveness of nucleoplasty, volumes between treatment and placebo groups were compared.

**Results:**

The average preoperative nucleus volume was 0.994 ml (SD: 0.298 ml). In the nucleoplasty group (n = 21) volume was reduced by an average of 0.087 ml (SD: 0.110 ml) or 7.14%. In the placebo group (n = 21) volume was increased by an average of 0.075 ml (SD: 0.075 ml) or 8.94%. The average nucleoplasty-induced volume reduction was 0.162 ml (SD: 0.124 ml) or 16.08%. Volume reduction in lumbar discs was significant in favor of the nucleoplasty group (p<0.0001).

**Conclusions:**

Our study demonstrates that nucleoplasty has a volume-reducing effect on the lumbar nucleus pulposus in an animal model. Furthermore, we show the volume reduction to be a coblation effect of nucleoplasty in porcine discs.

## Introduction

In the past numerous intradiscal therapies were used to treat discogenic pain and symptomatic disc herniation by performing indirect decompression via disc volume reduction [1]. Many studies were published on the clinical results of chemonucleolysis with different substances [2], [3] and percutaneous laser disc decompression (PLDD) [4]. For some procedures, efficacy has been investigated [1], [5]. The mechanism of action, however, has not always been clarified for every treatment form. Some treatments such as enzymatic chemonucleolysis have been abandoned due to their side effects [6]. In the mid-1980s various forms of laser disc decompression (PLDD) were established [7]. The clinical results reported later were in part positive [4], and, unlike chemonucleolysis, PLDD was reported to have an extremely low complication rate [8], [9]. In July 2000 the Food and Drug Administration (FDA) approved nucleoplasty with coblation for use in the USA [9]. Since then, some studies on the clinical efficacy of this procedure for the lumbar spine have been published [9]. The procedure is safe and has come to be used on the cervical spine as well [10]. Chen et al. made individual measurements to experimentally demonstrate a reduction in intradiscal pressure as a potential underlying mechanism [11]. Various authors have pointed to the histological effects of nucleoplasty [12], [13]. Based on experiences with other intradiscal procedures, we may assume that volume reduction has an effect on discal decompression as well [14]. In the assessment of physiologically and pathologically altered discs, magnetic resonance imaging (MRI) is the gold standard [15], [16]. MR scanners with field strengths of 1.5 and 3 Tesla have become established for clinical use [17], and for special investigations, high-resolution 7-Tesla ultrahigh-field MRI has recently become available [18]. MRI has also been shown to be well suited for the measurement of discal tissue volume [19]–[21].

This study explores volume-reducing effects of nucleoplasty in a model that is based on volumetry of the nucleus pulposus in pig discs in vitro, derived from 7.1-Tesla MRI data sets. To the best of our knowledge, lumbar postnucleoplasty volume reduction has not been investigated and published in the English literature.

**Figure 1:**
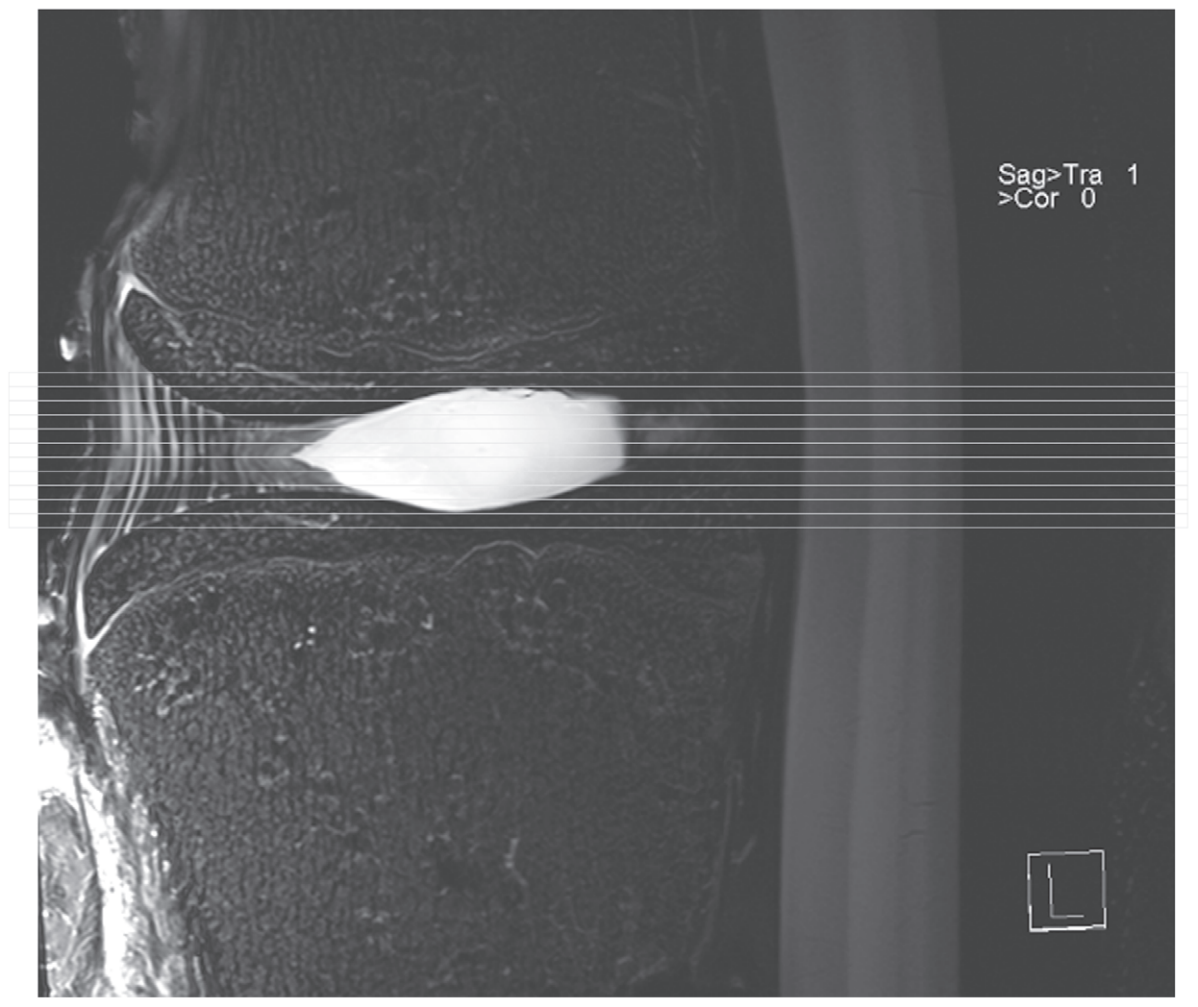
Sagittal images of lumbar motion segments showing axial stack. The axial images were acquired without gaps, parallel to the disc.

## Materials and Methods

### Specimens

For our study we used the ex vivo lumbar spines (L3 to L6) of 21 freshly butchered domestic German native breed pigs (mean age: 11.3 months; range 8–18 months). [22] Preinterventional MRI and treatment of dissected spines were performed immediately after butchering the animals. Discs that showed macroscopic signs of injuries from butchering, transport, or preparation were excluded from the study (n = 1).

**Figure 2:**
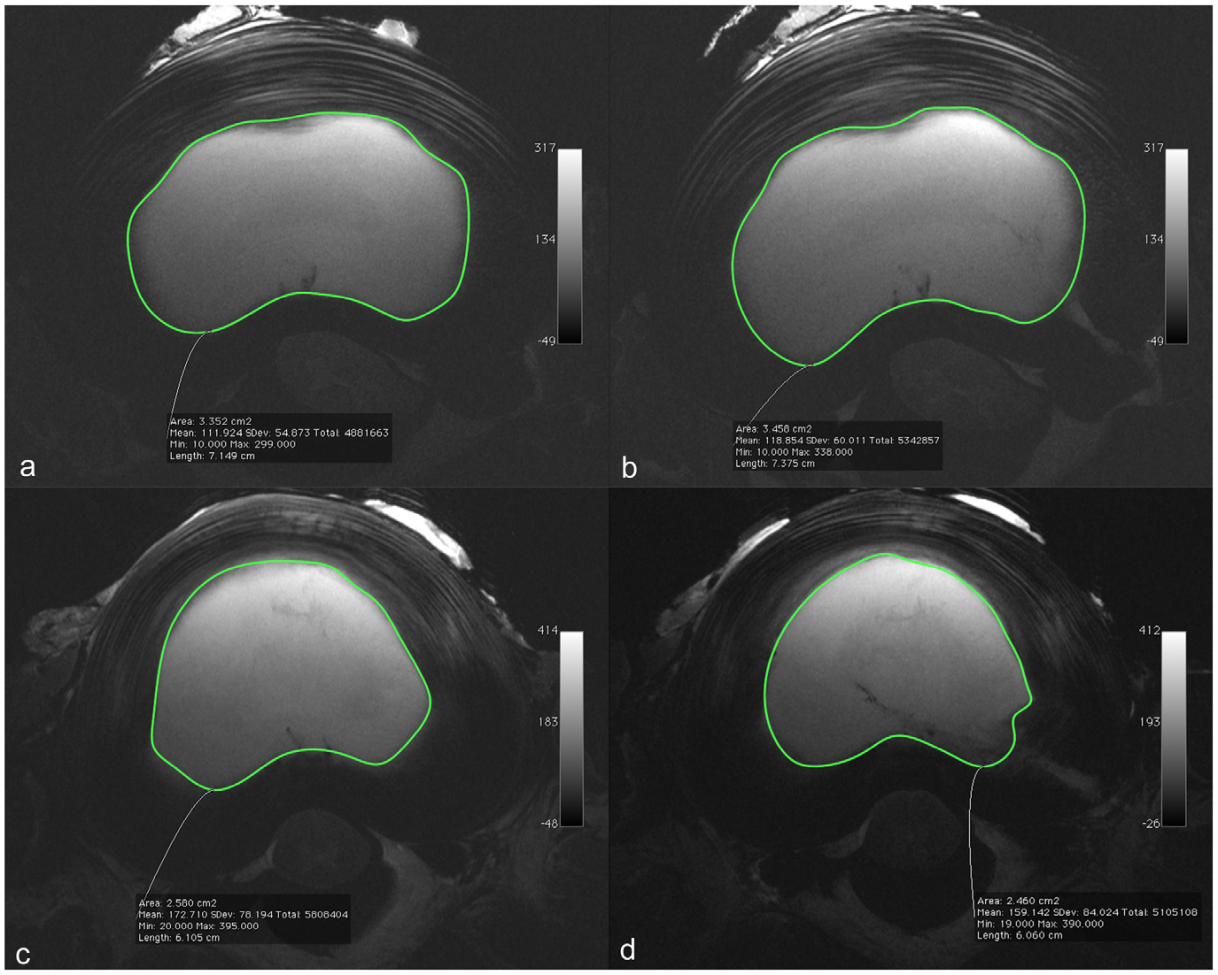
(a–d). T2-weighted, axial images of 6- and 9-month old experimental discs; the hyperintense nucleus pulposus and the surrounding inhomogeneous hypointense annulus fibrosus are depicted. (a,c): Preoperative images with outlining of the nucleus pulposus surface for volume calculation. (b,d) Postoperative images (b) (placebo group) with outlining of the nucleus pulposus (c): (nucleoplasty group) with outlining of the nucleus pulposus. Both postoperative images show the recognizable channel at 4–5 o’clock on the left, hypointense in relation to the nucleus pulposus and to the annulus fibrosus, hyperintense.

### Image Data Sets

Imaging was performed on a 7.1-Tesla ultrahigh-field MRI system (ClinScan, Bruker Bioscan GmbH, Ettlingen, Germany). The discs were imaged using a 1-channel rat brain coil. Before treatment the outside of each disk was marked so that it would be possible to compare the position of the preoperative disc in relation to the coil with the postoperative position. To calculate the volume of the nucleus pulposus, axially oriented, gapless, T2-weighted turbo spin-echo sequences (TR: 2000 ms, TE: 42 ms, slice thickness 0.7 mm, field of view 45×45 mm, 512×512 pixels) with an acquisition time of 7:30 minutes were obtained (Fig. 1).

**Figure 3:**
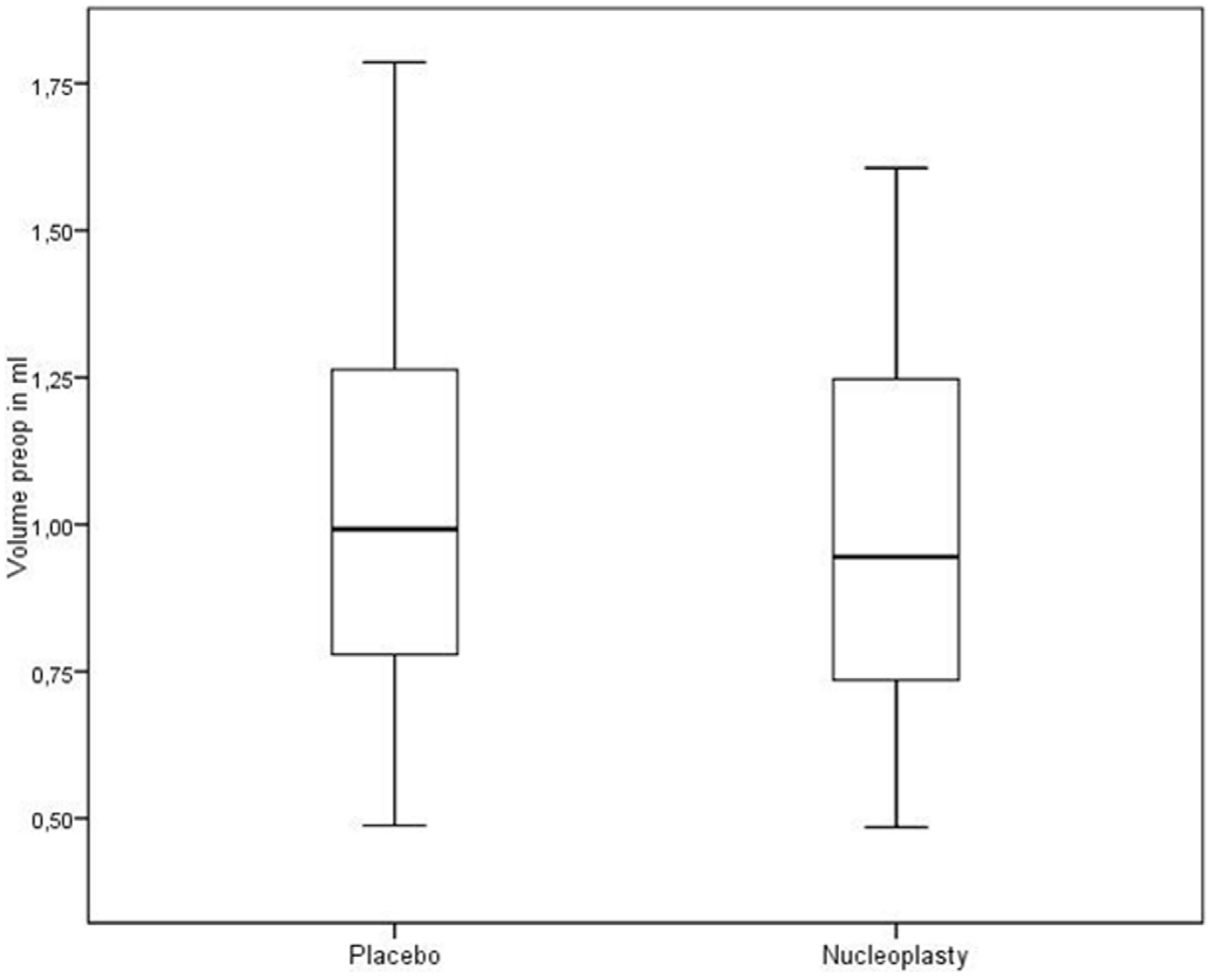
Initial volume of the nucleus pulposus in the independent lumbar spine groups (n = 21 in each) in a box-whisker plot, showing the 25% (lower box end), 50% (mark inside box) und 75% quartiles (upper box end). The whiskers represent the smallest and largest values within 1.5 × interquartile range. The group differences were not statistically significant (p = 0.919).

**Figure 4:**
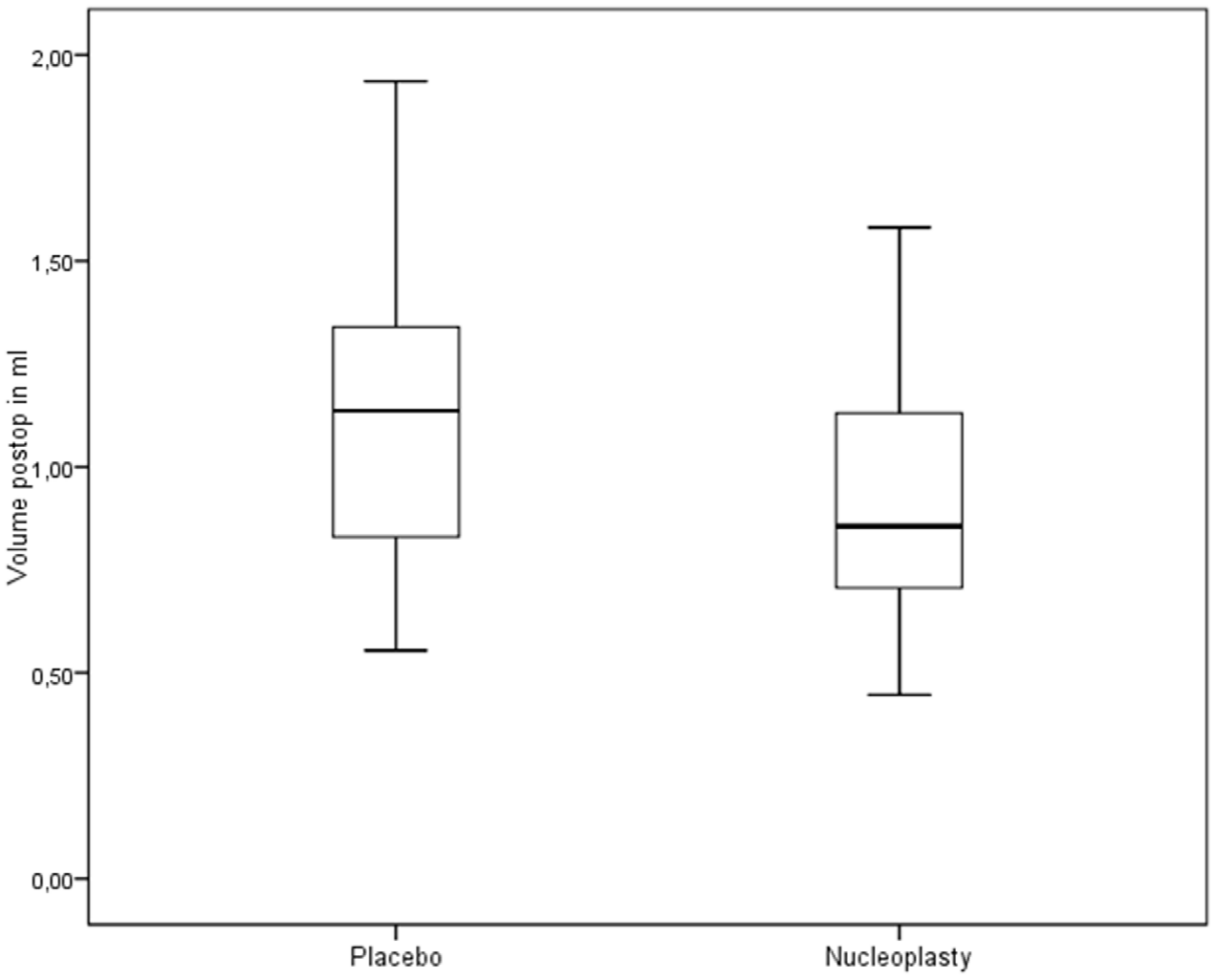
Postoperative volumes in the independent placebo group and nucleoplasty group (n = 21).

**Figure 5:**
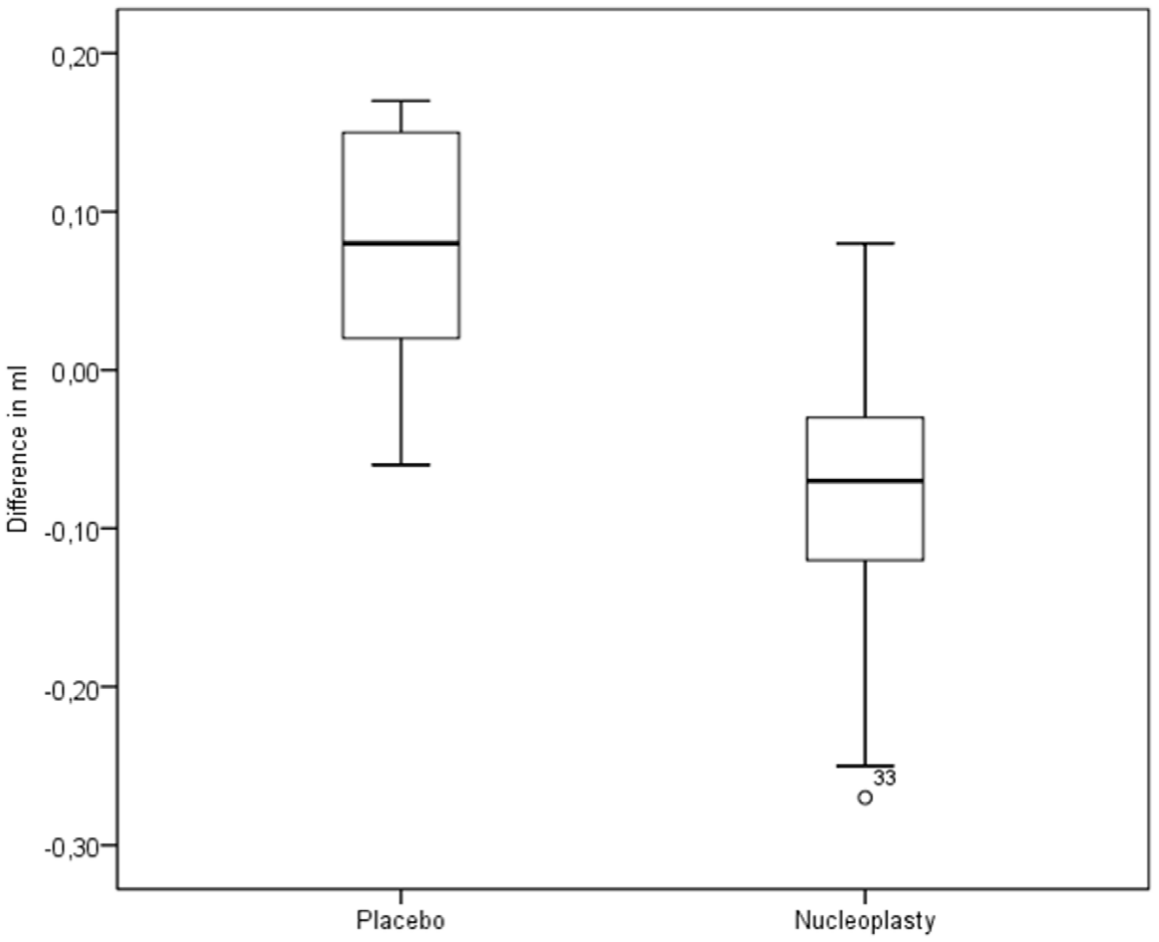
Difference between the initial and postoperative volumes, both in the independent placebo group and in the nucleoplasty group (n = 21), with volume increase in the placebo group and volume reduction in the nucleoplasty group (_°_- markers denote “mild” outliers within 3 × interquartile range).

### Volumetry

MRI images were viewed and interpreted the OsiriX® image viewing and processing software (Version 3.6.1, 32-bit, http://www.osirix-viewer.com) [23]. Volumetry was performed semi-automatically. We manually outlined the junction of the nucleus pulposus and the anulus fibrosus on the first and last slices of the axial series. The software program then used these to calculate the surface areas of the slices within the consecutive gapless series, and final corrections were made manually. Volume calculation was performed automatically in OsiriX® by multiplying the surface areas with the slice thickness. Volume measurements were carried out the same way before and after treatment or placebo treatment (Fig. 1, Fig. 2).

**Table 1:** Summary of the independent results of changes in lumbar intradiscal volume after nucleoplasty, compared to the placebo group.

Cohort	Volume change absolute		p	Volume change relative		P
Group	Nucleoplasty (n = 21)	Placebo (n = 21)		Nucleoplasty (n = 21)	Placebo (n = 21)	
Result	−0.087 ml (SD:0.110)	+0.075 ml (SD:0.075)	<0.0001	−7.14%	+8.94%	<0.0001

**Table 2:** Evaluation of intradiscal volume changes in the lumbar spine for independent pairs for placebo versus nucleoplasty (n = 42).

	Mean	Median	SD	Range	Interquartilerange	p
Δ V absolutein ml	0.162	0.140	0.124	0.536	0.114	<0.0001
Δ V relativein %	16.08	15.52	12.240	47.010	12.420	<0.0001

### Nucleoplasty

Nucleoplasty was performed ex vivo using the ArthroCare System 2000 (Fa. ArthroCare Deutschland, Remscheid, Germany) with control unit, foot switch, patient cable, and Convenience Pack (DLR SpineWand and sterile 17-Gauge Crawford needle (6“) with mandrin). In the nucleoplasty group the coblation current was applied to six channels for 10 seconds each with an application field of 360°. In the placebo group an identical protocol was followed, except that no current was applied. The orthopedic surgeon performing nucleoplasty was blinded to the type of treatment (with coblation current versus without (treatment versus placebo group)). For an analysis of the effectiveness of nucleoplasty, volumes were compared between the treatment and placebo groups.

### Data Selection

A total of 42 discs, 21 in the treatment group and 21 in the placebo group, were measured and used for the independent statistical analysis. Additionally, only one neighboring disk pair (L3/L4 and L4/L5 for example) per spine was considered. Spines were assigned to either group using the SAS randomization program. Independent value pairs were chosen randomly in order to minimize the potential for systematic bias in the sample (disc disease in one of the pigs for example).

### Statistics and Initial Tests of the Procedure

For statistical analysis the Wilcoxon test for independent samples (nonparametric) was applied. Also employed was the software SAS 9.1 TS (XP_PRO Windows NT-Server Version, Cary, North Carolina, USA). Calculated differences were considered significant when the probability of error remained below 5% (p<0.05).

The reproducibility and accuracy of volumetric measurements were evaluated by determining intra- and interrater variability. This was done by repeated measurement of 20 preoperative nuclei pulposi after training of the raters and before the actual study started. In the actual study, the raters were blinded to the group and all other data. Pre- versus postprocedure volume data were matched after completion of the entire experiment. Intra- and interrater variability was determined using Bland-Altman analysis (MedCalc, Mariakerke, Belgium). Statistical parameters were calculated, and graphic analysis was performed. Analysis of both intrarater and interrater variability showed very good results without major variations. Systematic errors were excluded.

### Ethics Statement

This study was carried out in strict accordance with the recommendations in the Guide for the Care and Use of Animal for Experiments of the National Institutes of Health. The protocol was approved by the Committee on the Ethics of Animal Experiments of the University of Greifswald. Ethical approval was not required because the pigs were not slaughtered for the purpose of our in vitro experiments. The experiment complied with the German Law on Animal Experiments. The animal parts were obtained from a research slaughterhouse and we obtained permission to use these animal parts from, FBN, Leibniz Institute for Farm Animal Biology.

## Results

During our ex vivo procedure there were no complications. All porcine discs were found to be normal without signs of injury or degeneration. In both postoperative groups we were able to track the channels created by the device. Since the nondegenerated nucleus pulposus is a soft, elastic, gelatinous structure, we did not identify regions with more marked volume reduction within the nucleus.

The average nucleus volume for all 42 discs examined in our study was 0.994 ml (SD: 0.298 ml).

The independent group yielded the following results. The initial volumes in the placebo and nucleoplasty groups were 1.003 ml (SD: 0.333 ml) and 0.998 ml (SD: 0.318 ml), respectively; the difference is not statistically significant (p = 0.919) (Fig. 3). The postoperative volumes in the placebo and nucleoplasty groups were 1.078 ml (SD: 0.350 ml) and 0.906 ml (SD: 0.287 ml) respectively; the difference is highly significant (p<0.0001) (Fig. 4). In the nucleoplasty group, the nucleus pulposus volume (pre- versus post-procedure) showed a significant reduction of 0.087 ml (SD: 0.110 ml) or 7.14% (p<0.0001). In the placebo group, the instruments applied produced a volume increase (pre- versus post-procedure) of 0.075 ml (SD: 0.075 ml) or 8.94% (p<0.0001) (Table 1), (Fig. 5). Overall, nucleoplasty decreased nucleus volume by 0.162 ml (SD: 0.124 ml) or 16.08% compared with placebo treatment (Table 2), (Fig. 5).

## Discussion

The clinical efficacy has been investigated for many of the intradiscal procedures used for treating symptomatic discs [1], [3]. However, for any clinical procedure to be truly accepted, an explanation of its mode of action is required. Moreover, the mechanism of action must be demonstrated in an experimental setting [24]. While the mechanism of action for some procedures, chemonucleolysis for example, is well established [25], [26], for others it has not been clearly demonstrated experimentally. Decompression is the desired effect of PLDD. This decompression has been attributed to biophysical factors [27] but also to a reduced intradiscal pressure [28], [29]. The efficacy of most intradiscal procedures is tied to a particular discal volume, limiting their usefulness in cases of height loss or disc degeneration. Our study used only spines from young pigs, which showed no degenerative changes in the preoperative MRI.

Yielding good clinical results, Nucleoplasty yields good clinical results and is considered a safe procedure with only a small number of side effects [1], [30]. In order to identify the potential risks of the procedure an elucidation of the underlying mechanism of action is necessary. Lee et al., in an animal experiment using sheep discs in vitro, showed that coblation, upon which nucleoplasty is based, does not produce any damage in the tissue outside of the plasma field that surrounds the electrode [12]. Chen et al., in their histological study on pig cadavers, also showed that nucleoplasty does not lead to any thermal damage in the surrounding tissue outside of the work field [13], [31]. It is known, however, that nucleoplasty produces temperatures of more than 45°, the temperature of neurodegeneration [31], and also that local temperatures of 60 to 65°C, or sometimes even higher, can be reached in the 3–4 mm margin surrounding the probe [32].

An experimentally demonstrated correlate of the efficacy of nucleoplasty is the reduction in pressure inside the disc [11]. Using three human cadavers, the authors showed that the intradiscal pressure reduction, produced by nucleoplasty, is dependent on the degree of degeneration within the disc. Podhajsky et al. used an in vivo animal model to also demonstrate an intradiscal pressure reduction after application of monopolar radiofrequency energy [33]. The pressure reduction effects persisted throughout the observation period of this experiment of 28 days. The reduction of nucleus pulposus volume we showed is the cause of the observed pressure effects. To our knowledge such quantitative lower lumbar volume effects have not been published before.

Coblation, induced by applying high-voltage bipolar radiofrequency current, can generate a plasma field, in which highly ionized particles, with enough energy to vaporize tissue, are generated [34]. Coblation has been applied in different conditions, such as tonsillectomy and chondroplasty, in which tissue degradation is indicated [35], [36]. Using 19 human cadavers, Kleinstueck et al. described the effects of coblation on the stiffness and degree of movement in the segments treated [37]. Chen et al. described the volume-reducing effects of nucleoplasty qualitatively, based on a microscopic analysis of histological specimens [11], [13]. The authors provide no quantitative data on the extent of ablation.

In our study we were able to show experimentally that, in a placebo-controlled setting, nucleoplasty reduces the initial volume of nucleus pulposus by a statistically highly significant degree. Our explanation for the volume increase in the placebo group is that the spine wand applied there pushed the nucleus aside. [21] In the nucleoplasty group, on the other hand, the suppressed volume was reduced with the coblation current.

The average nucleoplasty-induced volume reduction (pre- versus post-procedure) in our animal experimental study was 0.087 ml (SD: 0.110 ml) or 7.14%. In a human disc a volume reduction of approx. 1 ml corresponds to a reduction of approx. 10 to 20% [14]. When considering the average total size, 0.994 ml, of the nuclei examined here, the reduction in volume produced by nucleoplasty is of a magnitude that can be therapeutically effective in treating herniated discs.

This ex vivo study clearly demonstrates that there is a volume reduction after nucleoplasty; however, it is not clear how this will change over time. This can only be investigated in an in vivo study.

### Conclusion

In this study, it was shown that nucleoplasty causes a volume reduction of the nucleus pulposus, suggesting volume reduction as a potential mechanism by which this procedure brings about clinically effective decompression. Our data demonstrate the volume-reducing effect of coblation in nucleoplasty. While this effect was demonstrated in an ex vivo experimental setting and remains to be verified in patients, it points to the potential benefit likely to be achieved in a clinical setting.
